# Indents

**Published:** 1909-06-15

**Authors:** 


### INDENTS.

☞ **Brief Articles of Technical or Scientific Value will receive notice and comment. Contributions invited.**

*Quick Method of Making Counter Die.* Take the impression with a compound composed of two parts plaster and one part moulder's sand. Dry the impression thoroughly and pour in the die metal as cool as possible. When the metal has crystallized remove the die from the impression. Soften some modeling compound and place in the bottom of a vulcanite flask. Press the die in the modeling compound, cool in cold water and separate. You are now ready to swage the metal plate. By this method lead counterdies are dispensed with, and as all the appliances are in the office it will cost nothing to try it.—Dr. A. S. Waltz in *The Dental Office and Laboratory.*

*Repairing a hole in a Crown.* It is often necessary to repair a large hole in the occlusal surface of a molar crown. This may be accomplished successfully in the manner described below. Remove the crown and scale off any cement that has adhered to the root, and drill out the cement in the crown. Replace the crown in position on the root and press wax into the opening in the crown, being careful to leave enough extending occlusally to take an imprint of the opposing cusps. Take a bite in this wax and remove crown with wax intact. Flow plaster into the crown and when it is set remove the wax. If the hole does not include too much of the occlusal service the operator may retain in his mind the approximate form by studying the impression in the wax before removing it. If the hole includes a large portion of the occlusal surface the cast inlay method may be adopted. In the first instance make a matrix of the cavity and flow gold into it, building it up to the occlusal form as accurate as you can. In the second instance cast a gold inlay, place it in the cavity and attach to margins with solder. This will provide bulk of metal to insure the crown from failure due to the first cause of the hole. The occlusal surfaces of gold crowns are usually made too thin. —G. W. J., *Dental Review.*

*Urine Analysis in Pyorrhea Alveolaris.* I have lately been impressed by the coincidence of pyorrhea alveolaris and sugar and albumin in the urine, and the disappearance of these symptoms when the condition of the teeth was cured. I am collecting two hundred cases of pyorrea alveolaris, with urine analysis before and after the pyorrhea is cured. I wish to ask other practitioners who are interested in this subject, to kindly send me any results which they have in the same line, that is, when they find sugar and albumin in the urine, will they have the teeth investigated and find out how many of such patients have pyorrhea alveolaris? This is a subject of interest to all of us, and I am sure the results will amply repay us for the trouble involved.—William Martin Richards, *Jour. Amer. Med. Association.*

*Temporary Teeth.* When we find cavities in the children's teeth, the question with us is, how shall they be treated, and what material used in filling them so that they will give the best service, performing their function properly? One says, never under any circumstances should a deciduous tooth be devitalized. Another says, devitalize and remove contents from pulp chamber and fill that portion with good antiseptic paste (he uses a mixture of zinc oxid, iodoform and oil of cloves). Abscessed decidiuous teeth require most attention, and where it is advisable to fill the roots, they should be sterilized preferably with equal parts of trikresol and formalin, and filled with a material which is absorbed with the roots, for any other material offers a source of irritation for recurrence of the pathological condition.

We find it is almost impossible to dry the root canals thoroughly, so it becomes necessary to use a material that will adhere to moist surfaces. Dr. Henry Ferris, of Brooklyn, suggests a filling material which he finds meets the requirements for a good root canal filling material in temporary teeth. The formula he uses is: Isinglass, dr. i; tannic acid, gr. i ss.; trikresol, m. iv; aqua distilled, dr. i. The mixture is heated to 100 degrees in a water bath. It becomes syrupy, and can be introduced into roots and covered with cement or gutta percha.—S. G. Walton, in *Items of Interest.*

*Retention of Lower Plates.* In order to secure atmospheric pressure on the lower plate we must proceed by a method that has been formulated with the idea of taking into consideration the various conditions with which we meet in different mandibles, for if we can make a plate for the lower jaw that will exclude the air from under it, there is no reason why atmospheric pressure will not act as well there as with the upper jaw.

I use, for taking impressions of these cases, Modeling Compound. This material is warmed to a consistency at which it will adapt itself readily to the shape of the mouth.

It is then placed upon the tray, put in the mouth, and pressed down almost as far as required. Then wait for a minute or two until it hardens slightly. During this interval of time I have my assistant direct a current of cold air into the mouth from a rotary fan in the dental engine, which procedure chills the outer layer of the compound and prevents it from flowing away from the jaw when pressure is put upon it. While the layer of impression material that is in contact with the tissues is still warm and plastic, the tray is pressed down just a little farther, using from five to ten pounds pressure, and is then held steadily—with about half the force used in pressing it down the second time—until it is quite hard. The tray is then removed and the cast is poured.

In order that we may know why we proceed in this manner, let us go over the taking of this impression again. In the first place, when the material is put in the mouth and pressed down, we get an ordinary impression. After that is allowed to stand for a short time, and the outside is chilled and pressed down a second time, the impression is changed from the ordinary one, but in what way? When pressing down on the tray the second time the hard ridge of the alveolus cuts just a little deeper into the impression material and remains practically unchanged, while the soft tissues down on the sides of the gums about where the border of the plate reaches are compressed slightly. When the plate is made on the cast from this impression, and put into the mouth, the margins of the plate are sealed all the way around, and this converts the whole lower surface of the plate into one great suction pad—if we may use that term.

One of the most difficult parts of the operation is the trimming of the plate to just that line that will best seal its borders, for if it be too long it will give the muscles an opportunity to displace it. If too short, it may allow air to pass in, and thus defeat our purpose. It is self-evident that the upper plate is retained by air-pressure against gravity, while the lower plate adds its own weight to that of the atmospheric pressure; and so, compared with the upper, the lower has twice its own weight to add to the atmospheric pressure, thus showing that it is a better subject to be retained by such pressure than is the upper plate.—Dr. D. H. Young, May *Summary*.

*The Dittmar cast Gold-Shell Crown* A band of 34 ga. 24-k. gold is made and accurately fitted to the prepared root in the usual way. Thirty-four gauge pure gold is used because it is thin enough and soft enough to be most accurately modeled to the form of the root and this is extremely important. If the stump has been trimmed up to a slight taper, as it shculd be, the 34 gauge gold will stretch a little and thus hug the root beneath the gum margins as clcse as can be.

This band is now slitted down the sides in numercus places equal in depth, so that when the divisions are turned in to the center, lapping cne over the other at that point, the top is closed. Or if preferred, the band may be capped by soldering cn a flat piece at the right height to allow fcr the addition of occlusal thickness and cusps. With this now accurately adjusted to the stump, add cn to the top enough modeling compound to get a good bite, together with contact of approximating teeth. The crown shculd come away with the mcdeling wax, then fitted exactly in place. If not, it must be removed frcm the stump and carefully adjusted in the compound.

Now, this shell should be filled up with the silicate casting investment and the rest with plaster as usual in making up articulating models. Having secured articulating mcdels, soften and remove the modeling ccmpound and then, with a small brush, oil the plaster margin in arcund the crown, also the sides of approximating teeth.

The next step after gold is entirely clean, is to build up a tcp and lateral contour with Taggart or other suitable inlay wax. This is readily done by having the wax melted in a little dish and applying it with a fine camel's hair brush to the thickness desired cn all sides and on top of the gold. Then by clcsing the articulator with the wax warmed the impress or bite is made of the occluding teeth, and the necessary carving and ccclusal shaping up may be done; also the contouring on all sides, painting on more wax or trimming away and smoothing and polishing up until a perfect tooth model is formed over the gold shell. None of the wax should impinge on the plaster. The oil is applied to prevent the wax that may accidently touch the plaster from sticking.

Now the adjoining teeth may be broken away, leaving the crown model standing alone The approximal surfaces should now be painted with a thin layer of wax to insure correct contact after finishing the gold. If this is not done the crown might be a little too narrow approximally. It is better to have too much than too little.

Now the smoother our model is finished and polished the better the cast will be. The model can now be sawed off from the plaster and is ready for investment after inserting the sprue at the most available point. Of course the shell remains in the mold and the molten gold takes the place of the disappearing wax.

When this crown is finished, it is about as perfect as can be made, having a very thin edge to pass between the gum margin with gradually thickening walls to and including the occlusal portion. Twenty-two carat gold should be used for the casting, as it is harder and more durable.

A crown made in this way contains a good deal more gold than a shell crown made of plate in the old way, it is worth more intrinsically, artistically and every other way, and a good price should be charged for it.

For bridge supports they are far superior in strength to the old style shell crowns that being thin-plate yield more or less under stress. Any cast crowns are superior in this respect. There is no springing and yielding in the heavy work that may be put upon them. Such a weakness in the usual shell crown has been the cause of many a failure in bridge work; and besides that, the lack of close adaptation to the shape of the root, which can be secured in this way. —R. B. Tuller, in *American Dental Journal.*

*National Dental Association Meeting—a Summary Report.* The thirteenth annual meeting of the National Dental Association was held at Birmingham, Ala., March 30th, to April 2nd inclusive, and was an especially interesting meeting in many respects.

In the first place the attendance was much larger than was expected in view of the fact of the much earlier date than heretofore, which necessarily prevented the National

Association of Dental Examiners and the National Faculty Association from meeting in conjunction therewith, but the advantage of an early meeting, when the Association goes south, is worthy of the most careful consideration.

The papers read before the general session were quite interesting and elicited enthusiastic discussion, while those read before the sections were well received and thoroughly discussed.

The president's address contained a number of timely suggestions dealing with the important questions before the profession to-day. The committee appointed to report on this address emphasized the necessity of a systematic effort to secure the co-operation of the various State Dental Societies along the line of broader National Dental Association, as follows: "We, your committee, urge the necessity of a consecutive and consistent effort on the part of the officers and members of the Association, to establish a loyal relation between our National body and various State Dental Associations throughout the country, and that especially the corresponding secretary, who is elected this year, shall undertake a systematic correspondence with the officers of said State Societies and where possible visit them as a representative of this Association and present the claims and advocate the interests of our National body."

The report of the committee on the *National Dental Journal* gave evidence of the interest taken and the time given to their work, and it was decided by the Association to begin the publication of such a Journal commencing with the October, 1910, issue. The interest manifested in this was such as to give evidence of general support and interest in a National Dental Journal.

The committee appointed last year on revision of Constitution and By-Laws made an extended report and presented a number of amendments embodying a liberal plan of reorganization. Copies, with the proposed changes, are to be printed and mailed to the membership at an early date, which will give ample opportunity to thoroughly understand same before final action is taken at the 1910 meeting.

One particular amendment presented by the committee, and which received much favorable comment, makes provision for State Dental Societies becoming component parts of the National Association, and when such societies join in a body, the dues of the membership of the State Society shall be two dollars a year, which entitles such members to the *National Dental Journal* without additional cost, while the subscription price of the *Journal*, to other than members of the Association, is two dollars. Thus , it will be seen that there is a general disposition to make our National Association a truly representative body of the dental profession; therefore, let us all do our part to accomplish this much desired end, thereby increasing our usefulness in many directions.

The legislative committee reported that every effort had been made to secure Army and Navy dental legislation, through the last congress, but that conditions arose, over which they had no control, which defeated their purposes.

A new legislative committee was appointed, consisting of Dr. Wm. Crenshaw, of Atlanta, Ga., and Dr. W. H. Des Ford, of Des Moines, Iowa, together with the vice-presidents, the two secretaries and the treasurer.

The Navy Dental Bill, introduced March 29, 1909, by Senator Charles Dick of Ohio, was endorsed, and a vote of thanks extended to the Senator, and others, for their generous support in our efforts to secure Army and Navy dental legislation. As an Ohioan, and a member of the present and past legislative committee, I am pleased to say that our Senators and Congressmen have been loyal to our cause, and our profession generally has responded promptly to our calls, for which I am deeply grateful.

Denver, Col., was selected as the place for the next meeting and the third Tuesday of July, 1910, the time of holding same. This place and date were unaminously chosen and everyone seemed particularly pleased with the idea of visiting Denver at that time of the year. Arrangements should be made for railroad rates providing for stop-over privileges, and a sufficient length of time to permit those attending to spend several days in and around Denver following the meeting, as this arrangement will insure more interest being taken in the sessions of the Association, instead of devoting time, during the meeting, to side attractions.

The officers are already at work arranging plans to make this the most successful meeting the Association has ever had.

The southern dentists, and especially the local committee, did everything possible to make our stay in Birmingham both pleasant and profitable. The courtesies of the Birmingham Club were extended to the members, and a barbecue was given at the Country Club Wednesday afternoon, followed by a dance at the Club in the evening.

Quite a number took advantage of the opportunity, in going or returning, to visit the historic battlefields around Chattanooga, and I can personally vouch for a most enjoyable day being spent there, by a good sized party, on our return trip from the meeting.—Dr. H. C. Brown, in *Summary*.

